# Determining the minimum inhibitory concentrations of pyrazinamide against *Mycobacterium tuberculosis* clinical isolates at a neutral pH of 6.8 using the broth microdilution method

**DOI:** 10.3389/fmicb.2025.1688772

**Published:** 2025-10-21

**Authors:** Maria Tamblin, Wanliang Shi, Liang Chen, Jessica Reynolds

**Affiliations:** ^1^Department of Medicine, Jacobs School of Medicine and Biomedical Sciences, University at Buffalo, Buffalo, NY, United States; ^2^PZA Innovation LLC, Baltimore, MD, United States; ^3^Division of Clinical and Translational Therapeutics, School of Pharmacy and Pharmaceutical Sciences, University at Buffalo, Buffalo, NY, United States

**Keywords:** *Mycobacterium tuberculosis*, pyrazinamide, susceptibility, minimum inhibitory concentration, broth microdilution method, phenotypic

## Abstract

**Introduction:**

Pyrazinamide (PZA) is a critical component of first-line tuberculosis (TB) treatment. Misdiagnosis of PZA resistance can lead to serious consequences, highlighting the need for accurate and reliable PZA susceptibility testing. While broth microdilution is a cost-effective and widely used method for determining the minimum inhibitory concentrations (MICs) of antibiotics, its current application for PZA has been limited by the requirement for acidic conditions in conventional *Mycobacterium tuberculosis* culture media.

**Methods:**

In this study, we determined the MICs of PZA against the clinical isolates of pyrazinamidase-positive *M. tuberculosis* at a neutral pH of 6.8 using a defined culture medium and the standard protocol of the broth microdilution method.

**Results:**

The results showed that PZA MICs could be reliably determined in *M. tuberculosis* clinical isolates, with values ranging from ≤12.5 to 100 μg/mL.

**Discussion:**

This approach overcomes the limitations of existing acidic pH-based PZA susceptibility tests and provides a reliable, accurate, cost-effective method for detecting improved PZA resistance. Implementing this method could significantly enhance TB treatment, resistance surveillance, and efforts to combat drug-resistant TB.

## Introduction

Pyrazinamide (PZA) is a key component of modern tuberculosis (TB) treatment regimens. It is particularly valued for its ability to shorten the duration of TB therapy from 9 to 12 months down to 6 months (Grosset, 1978; Zhang et al., 2013; World Health Organization, 2017). Additionally, it plays a crucial role in treating multidrug-resistant TB (MDR-TB) and is a key element in short-course treatment regimens. Misdiagnosis of PZA susceptibility can lead to ineffective treatment, poor outcomes, and the development of drug-resistant strains (Yee et al., 2012; Budzik et al., 2014; Shouyong Tan et al., 2016). The rising prevalence of PZA resistance threatens treatment success and highlights the need for reliable drug susceptibility testing (DST) (Yee, 2006; Mphahlele et al., 2008; Whitfield et al., 2015).

There are two major approaches to DST: phenotypic and genotypic. Phenotypic DST involves culturing *Mycobacterium tuberculosis* clinical isolates and assessing growth inhibition in the presence of antibiotics, with a control group that is not exposed, under controlled laboratory conditions. The results are interpreted based on established clinical breakpoints [such as those from the Clinical and Laboratory Standards Institute (CLSI) and the European Committee on Antimicrobial Susceptibility Testing (EUCAST)] to classify clinical isolates as susceptible (S), intermediate (I), or resistant (R). Phenotypic DST is considered the gold standard due to its ability to quantify drug susceptibility with a standardized protocol closely related to treatment outcomes (Canetti et al., 1963). In contrast, genotypic DST has emerged from advancements in molecular technology, improving the understanding of the genetic mechanisms underlying drug resistance (Cirillo et al., 2017). Genotypic DST methods, which include techniques such as polymerase chain reaction (PCR) and sequencing of target genes or the whole genome, have gained prominence for rapidly predicting drug-resistant strains. These methods can detect mutations associated with drug resistance at the time of TB diagnosis; however, a key drawback is their dependence on existing knowledge of resistance mechanisms and access to advanced bioinformatics technology (Doyle et al., 2020; Davies et al., 2023).

PZA DST presents unique challenges. Unlike other TB drugs, PZA exhibits no activity against *M. tuberculosis* at neutral pH in conventional culture media, requiring either acidic (pH 5.0–5.5) or alkaline (pH 8.5) conditions for *in vitro* activity (McDermott and Tompsett, 1954). The current phenotypic susceptibility test for PZA is performed at pH 5.9 using a macrodilution method to detect PZA resistance by evaluating whether the strain can grow at a critical concentration of PZA (CLSI, 2011; CLSI, 2018). However, this method has several significant limitations, including unreliable results and high rates of false resistance—even after adhering to a substandard DST protocol (Siddiqi, 2004; World Health Organization, 2007; Woods et al., 2011). Moreover, the requirement for acidic pH prevents the use of microdilution or agar proportion methods, which are routinely employed for determining the minimum inhibitory concentrations (MICs) of other TB drugs (Woods et al., 2011; Cirillo et al., 2017; World Health Orginization, 2018). As a result of these technical challenges, phenotypic PZA susceptibility testing is not routinely performed in clinical laboratories.

PZA is a prodrug that requires activation by *M. tuberculosis* enzyme pyrazinamidase (PZase), which is encoded by the *pnc*A gene (Konno et al., 1967; Scorpio and Zhang, 1996). Although loss of PZase activity mutations in *pnc*A is a well-documented mechanism of PZA resistance, certain polymorphisms and some newly emerging mutations do not confer PZA resistance (Zhang et al., 2013; Yadon et al., 2017; World Health Orginization, 2023). Another limitation of PZase activity testing or *pnc*A gene sequencing for detecting PZA resistance is its inability to capture resistance mechanisms by PZA’s target gene mutations (Shi et al., 2011; Shi et al., 2014; Gopal et al., 2016; Yee et al., 2017; Zhang et al., 2017; Shi et al., 2018). These complexities make predicting PZA susceptibility one of the most technically challenging aspects among TB drugs, as extensively discussed in recent white papers (Association Of Public Health Laboratories, 2016, 2022).

Given these limitations, approaches for accurate and reliable PZA susceptibility testing are urgently needed. Recent studies have demonstrated that PZA exhibits anti-TB activity at a neutral pH of 6.8 in defined media, particularly against laboratory strains of *M. tuberculosis* (Shi, 2021). This discovery opens up the possibility for standardized PZA MIC testing using the broth microdilution method at neutral pH, which is optimal for *M. tuberculosis* growth, thereby potentially overcoming the challenges associated with acidic testing conditions. The MIC is defined as the lowest concentration of an antimicrobial agent that prevents the growth of a microorganism under standardized incubation conditions and is typically expressed in μg/mL. Depending on the degree of growth inhibition, which can be determined by colony-forming unit (CFU) counting, MIC values may also be reported as MIC₉₀ (90% inhibition) or MIC₅₀ (50% inhibition) (Canetti et al., 1963; Canetti et al., 1969; Sirgel et al., 2009). In clinical practice, the broth microdilution method typically relies on semi-quantitative measurements, such as inhibition of visible growth, to determine MICs. For a given drug, MIC values may vary depending on the assay used; however, a lower MIC value consistently indicates greater potency (Turnidge and Paterson, 2007; World Health Orginization, 2021, 2022). For mycobacteria, as an alternative, fluorescence-based growth indicators, such as oxygen sensors, have been employed to monitor microbial growth (Stitt et al., 2000; Hutter and John, 2004; Siddiqi and Rüsch-Gerdes, 2006). The sensor has been used for MIC determination with the broth macrodilution method (Siddiqi and Rüsch-Gerdes, 2006). Incorporating fluorescence-based growth indication further enhances this method by addressing common issues with visual readings, such as slow growth, clumping, and poor turbidity resolution, which often compromise the reliability of DST. In this study, we evaluated a broth microdilution method based on inhibition of visible growth at neutral pH (6.8) using fluorescence-based growth indication to determine the MICs of PZA against a panel of *M. tuberculosis* clinical isolates with confirmed PZase activity. Our findings demonstrate the feasibility and reliability of this approach, which may serve as a valuable tool in routine PZA susceptibility testing and resistance surveillance.

## Materials and methods

### *M. tuberculosis* clinical isolates

A total of 25 non-resistant *M. tuberculosis* clinical isolates with confirmed positive PZase were obtained from BEI Resources (Table 1) and included in this study. The reference strain *M. tuberculosis* H37Rv ATCC 27294 was used as a control. In addition, three PZA-resistant strains, *M. tuberculosis* Z6, Z78, and ORA136—all derivatives of *M. tuberculosis* H37Ra—were tested. The strains carry mutations in PncA (L159P or C138R) or PanD (E126*).

**Table 1:** Characteristics and PZA MICs of *M. tuberculosis* clinical isolates and reference strains used in this study.

Strain name	BEI Catalog No	Isolated from tissue	Year	Geographic information	PZA MIC (μg/mL)	Turnaround time (day)
*M. tuberculosis* HN1135	NR-20786	Human pulmonary tissue	1997	Texas, USA	≤12.5	13
*M. tuberculosis* HN1150	NR-20787	Human pulmonary tissue	1997	Texas, USA	50	23
*M. tuberculosis* HN1151	NR-20788	Human pulmonary tissue	1997	Texas, USA	50	17
*M. tuberculosis* HN1175	NR-20785	Knee	1997	Texas, USA	≤12.5	15
*M. tuberculosis* HN1210	NR-20790	Human pulmonary tissue	1997	Texas, USA	25	17
*M. tuberculosis* HN133	NR-20794	Human pulmonary tissue	1995	Texas, USA	25	23
*M. tuberculosis* HN1380	NR-20789	Lymphatic tissue	1998	Texas, USA	≤12.5	15
*M. tuberculosis* HN1430	NR-18993	Lymphatic tissue	1998	Texas, USA	100	20
*M. tuberculosis* HN1475	NR-18995	Human pulmonary tissue	1998	Texas, USA	25	13
*M. tuberculosis* HN1500	NR-20793	Human pulmonary tissue	1997	Texas, USA	≤12.5	21
*M. tuberculosis* HN1523	NR-20791	Human pulmonary tissue	1998	Texas, USA	25	23
*M. tuberculosis* HN1744	NR-20792	Human pulmonary tissue	1998	Texas, USA	25	13
*M. tuberculosis* HN2136	NR-20782	Human pulmonary tissue	1999	Texas, USA	25	15
*M. tuberculosis* HN2163	NR-20784	Human pulmonary tissue	1999	Texas, USA	≤12.5	31
*M. tuberculosis* HN2206	NR-20779	Human pulmonary tissue	2000	Texas, USA	≤12.5	10
*M. tuberculosis* HN2492	NR-20780	Human pleural fluid	2000	Texas, USA	50	13
*M. tuberculosis* HN268	NR-20796	Human pulmonary tissue	1995	Texas, USA	≤12.5	23
*M. tuberculosis* HN2863	NR-20777	Human Pulmonary Tissue	2001	Texas, USA	≤12.5	10
*M. tuberculosis* HN2925	NR-20781	Human pulmonary tissue	2008	Texas, USA	≤12.5	13
*M. tuberculosis* HN3171	NR-20776	Human pulmonary tissue	2002	Texas, USA	50	13
*M. tuberculosis* HN4112	NR-20775	Human pulmonary tissue	2003	Texas, USA	≤12.5	10
*M. tuberculosis* HN4143	NR-20783	Human pulmonary tissue	2003	Texas, USA	≤12.5	15
*M. tuberculosis* HN4687	NR-19033	Human pulmonary tissue	2007	Texas, USA	25	13
*M. tuberculosis* HN4689	NR-19035	Human pulmonary tissue	2006	Texas, USA	25	13
*M. tuberculosis* HN499	NR-20795	Human pulmonary tissue	1996	Texas, USA	25	23
*M. tuberculosis* H37RV					25	13
*M. tuberculosis* Z6					800	13
*M. tuberculosis* Z78					800	17
*M. tuberculosis* ORA136					800	17

### Broth microdilution method

**Figure 1:**
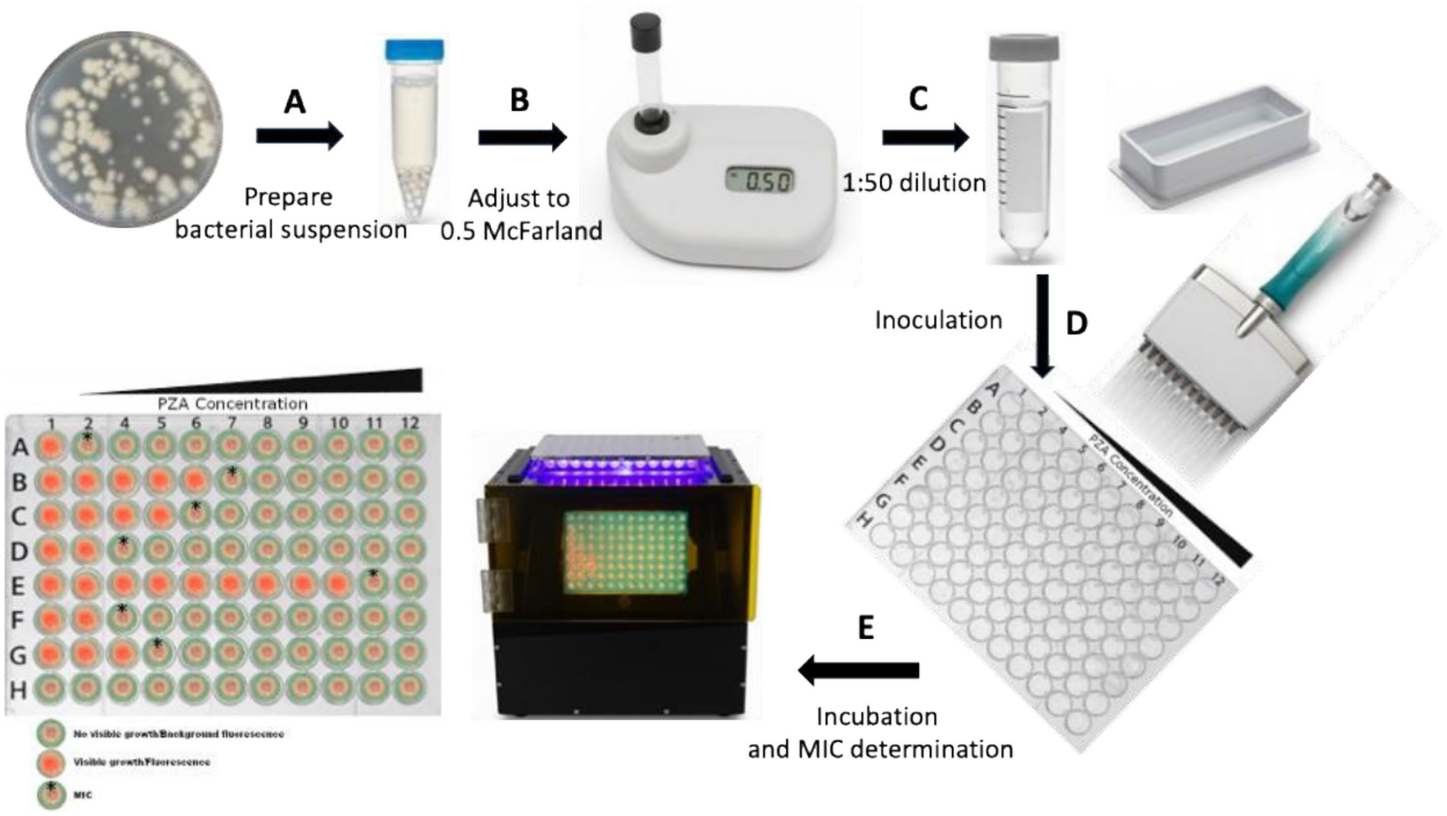
Schematic diagram of PZA MIC determination in a dry-format 96-well plate using the broth microdilution method. **(A)** Fresh cultures from 7H10 or 7H11 agar plates are harvested to prepare the initial bacterial suspension. **(B)** The suspension is adjusted to a turbidity equivalent to 0.5 McFarland using a McFarland reader. **(C,D)** The 0.5 McFarland suspension is diluted 1:50 to obtain the final inoculum, and 200 μL is dispensed into each well of the dry-format PZA DST 96-well plate. (E) The plate is incubated at 37 °C, and the results are read using a plate reader (black box, center) to determine the MIC. Column 1 contains PZA-free medium, while columns 2–12 contain increasing PZA concentrations, with column 12 having the highest concentration. Each strain is inoculated into designated rows, while row H serves as a negative control (medium only, no inoculum) to monitor contamination. Bacterial viability is confirmed by visible growth/fluorescence in the PZA-free control wells, which must be significantly stronger than the negative control wells. The MIC is defined as the lowest PZA concentration at which no visible growth or fluorescence is observed, provided that the PZA-free control wells show growth (red fluorescence). (Bacterial growth is indicated by red fluorescence, while absence of growth is indicated by background color. MIC wells are highlighted with a star on the left schematic of the 96-well plate).

PZA susceptibility testing was conducted using a dry-format 96-well PZA DST plate (pH 6.8) (PZA Innovation LLC, United States), as previously described (World Health Orginization, 2022). Briefly, fresh cultures from 7H10 or 7H11 agar plates, no older than 4 weeks, were initially suspended in PZA DST buffer containing 5.0 mL/L glycerol and 0.025% (vol/vol) Tween 80 (Figure 1A). The suspension was adjusted to a turbidity equivalent to a 0.5 McFarland standard and then diluted 1:50 in the same buffer to prepare the final inoculum (Figures 1B,C). Each well of the PZA DST plate was inoculated with 200 μL of the diluted bacterial suspension (Figure 1D), resulting in a final inoculum at a density of ~1.0–5.0 × 10^5^ CFU/mL per well (Woods et al., 2011; CLSI, 2018; Schön et al., 2020; World Health Orginization, 2022). To determine accurate MICs of PZA for each isolate, PZA was tested across 11 concentrations: 12.5, 25, 50, 75, 100, 125, 150, 200, 300, 400, and 800 μg/mL, along with a no-drug control. Plates were sealed with sterile sealing foils (Cat# FSC-25, Excel Scientific, Inc.) and incubated at 37°C. Plates were read on days 1 and 10, and every 2–3 days thereafter, using a mirrored box under natural light to assess visible growth, or under a dark background with excitation from a 395–440 nm light-emitting diode (LED) light to detect fluorescence. For wells containing an oxygen sensor, fluorescence was generated by an oxygen-quenched fluorochrome—*tris* (4,7-diphenyl-1,10-phenanthroline) ruthenium (II) chloride—embedded in silicone at a concentration of 68 μg/mL. During bacterial growth, oxygen is consumed and replaced by carbon dioxide, resulting in a depletion of free oxygen. As quenching is relieved, the fluorochrome emits visible fluorescence under ultraviolet (UV) light, directly reflecting bacterial growth (Stitt et al., 2000; Siddiqi and Rüsch-Gerdes, 2006). A test was considered valid if there was no contamination, if the no-drug control wells demonstrated adequate growth, and if the MICs of control strains were within expected reference ranges. The MIC was defined as the lowest concentration of PZA at which no visible growth or fluorescence was observed, in contrast to visible growth or strong fluorescence in the no-drug control wells (Figure 1E).

## Results

### Agreement between visible growth and fluorescence readouts for PZA MIC determination at neutral pH (6.8) using the broth microdilution method

To evaluate the consistency of visible growth and fluorescence readouts for PZA MIC determination, a broth microdilution assay was performed using the same batch of diluted bacterial suspension of the reference strain *M. tuberculosis* H37Ra and PZA DST plates with and without an oxygen sensor as a mycobacterial growth indicator. Growth was assessed on day 16 using a mirrored box (Supplementary Figures S1A, B). Visible growth attached to the bottom of the wells was observed in the no-PZA control wells, while partial growth was noted at 12.5 μg/mL. No visible growth was shown in the wells at 25 μg/mL (highlighted in the yellow box, Supplementary Figure S1A), as the no inoculum control (Supplementary Figure S1B) This concentration was defined as the MIC of PZA as 25 μg/mL based on visible growth.

Fluorescence was assessed on day 10 using a 410 nm LED light source in a mirrored box. Fluorescence was significantly increased in the no-PZA control well and diminished at 12.5 μg/mL, indicating slight growth as reduced oxygen consumption, no fluorescence was detected at 25 μg/mL (Supplementary Figure S1C) and the no inoculum control (Supplementary Figure S1D), indicating complete inhibition of growth as the absence of oxygen consumption and consistent with the turbidity results, thereby confirming the MIC of PZA as 25 μg/mL (highlighted in the yellow box, Supplementary Figure S1C).

These findings demonstrate complete concordance between visible growth- and fluorescence-based MIC determination for *M. tuberculosis* H37Ra using PZA DST plate under neutral pH conditions. Replicate experiments yielded consistent MIC values, supporting the robustness and reproducibility of both detection methods for PZA susceptibility testing (data not shown). All assays were considered valid, as no growth was observed in the no-inoculum control plates, confirming the absence of contamination.

### PZA MIC determination for *M. tuberculosis* clinical isolates using the dry-format PZA DST plate with the mycobacterial growth indicator

To evaluate the feasibility and reliability of a neutral pH dry-format PZA DST plate, we tested 25 *M. tuberculosis* clinical isolates obtained from BEI Resources. These isolates were originally collected from human pulmonary (21/25) or extrapulmonary samples (4/25) in Texas, USA, between 1995 and 2008, and were cataloged as non-resistant strains with positive PZase activity. Additionally, the reference strain H37Rv, as well as the PZA-resistant mutant strains Z6, Z78, and ORA136 derivatives from *M. tuberculosis* H37Ra, were tested. The MICs of PZA for the tested clinical isolates ranged from ≤12.5 to 100 μg/mL (Table 1 and Supplementary Figure S2). Notably, 20 out of 25 isolates (80%) exhibited a PZA MIC of ≤25 μg/mL (e.g., see Supplementary Figures S2A,B,D,F,-H), indicating high susceptibility under the neutral pH 6.8 condition. An additional 4 isolates (16%) showed intermediate MICs of 50 μg/mL (for example, see Supplementary Figure S2E), while only 1 isolate (4%) had an MIC of 100 μg/mL (for example, see Supplementary Figure S2C) Figure S2C), indicating a likely reduced susceptibility of PZA. The H37Rv reference strain displayed a PZA MIC of 25 μg/mL (Supplementary Figure S2H. In contrast, the PZA-resistant strain Z6, harboring a PncA L159P mutation, exhibited a markedly elevated MIC of 800 μg/mL (Supplementary Figure S2I). Similarly, the PZA-resistant strains Z78 and ORA136 also showed PZA MIC of 800 μg/mL, confirming their resistance phenotype under these assay conditions. CFU counts from a 0.5 McFarland bacterial suspension were measured for strain HN2925 (NR-20781) and reference strain H37Ra. CFU counted on 7H10 agar were 2.24 × 10⁷ and 1.72 × 10⁷ CFU/mL, respectively, at a standard inoculum size for reliable anti-TB drugs, such as rifampin and isoniazid, and MIC determination using the broth microdilution method.

The turnaround time for obtaining interpretable MIC results ranged from 10 to 31 days. Most isolates (76%) produced valid readouts within 15 days, demonstrating the assay’s potential for timely reporting in clinical or research settings, as well as other settings (Hall et al., 2012; Yu et al., 2016; CRyPTIC Consortium, 2022; Puyen et al., 2022).

## Discussion

This study demonstrates that PZA MICs can be reliably determined using a broth microdilution method at neutral pH (6.8), a significant step forward in addressing the limitations of current PZA DST methods. Conventional PZA DST’s acidic condition, while required for PZA activation, suppresses *M. tuberculosis* growth and has been associated with high rates of false resistance and poor reproducibility even under standardized conditions (Association Of Public Health Laboratories, 2016; Morlock et al., 2017; World Health Orginization, 2018; Köser et al., 2020; Mok et al., 2021; Association Of Public Health Laboratories, 2022; World Health Orginization, 2022). Reported false resistance rates can exceed 20% under current acidic PZA testing conditions (Zhang et al., 2002; Piersimoni et al., 2013; Mustazzolu et al., 2019), which significantly undermines test reliability. Moreover, these methods typically detect resistant populations only at ≥10%, far above the 1% threshold recommended for most antibiotic DSTs to prevent treatment failure (Siddiqi and Rüsch-Gerdes, 2006; Woods et al., 2011; World Health Orginization, 2018). Due to PZA’s acidic activity requirement, CLSI, EUCAST, and WHO currently do not recommend the broth microdilution for PZA testing in *M. tuberculosis* (Simons and van Soolingen, 2011; Cirillo et al., 2017; World Health Orginization, 2018; Woods et al., 2019; World Health Orginization, 2022). These limitations have contributed to the exclusion of PZA phenotypic testing from routine clinical practice (Canetti et al., 1963; Canetti et al., 1969; CLSI, 2011; World Health Orginization, 2018).

Our findings show that PZA retains measurable activity at neutral pH in a defined broth system, and that MICs can be consistently determined using a standardized inoculum and broth microdilution method protocol. The inoculum size used (~1–5 × 10^5^ CFU/mL in 200 μL per well) falls within well-established guidelines for DST in *M. tuberculosis* (Bernardelli et al., 2004; Penuelas-Urquides et al., 2013). Fluorescence-based oxygen sensors, which detect metabolic activity through oxygen consumption, provide a robust alternative to conventional optical density at 600 nm (OD₆₀₀) measurements for quantifying mycobacterial growth (Stitt et al., 2000; Hutter and John, 2004; Siddiqi and Rüsch-Gerdes, 2006). While OD₆₀₀ is a reliable indicator of cell density in well-dispersed organisms such as *Escherichia coli* and *Staphylococcus aureus* (Hinds and Peterson, 1963; Tiwari et al., 2023), it is unsuitable for *M. tuberculosis*. The hydrophobic, mycolic acid–rich cell wall of *M. tuberculosis* promotes grainy, flocculent aggregation and cording in liquid media, resulting in a non-linear relationship between OD₆₀₀ readings and viable cell density (Dubos and Middlebrook, 1947; Parish and Kumar, 2021). To overcome these limitations, fluorescence-based growth indicators can be incorporated into a 96-well plate format, enabling the development of a high-throughput, automated antimicrobial susceptibility testing (AST) platform (Tiwari et al., 2023). All 25 clinical isolates tested in this study had confirmed PZase activity. Among them, MICs ranged from ≤12.5 to 100 μg/mL, indicating a spectrum of PZA susceptibility. The susceptible reference strain H37Rv had an MIC of 25 μg/mL, while the PZA-resistant Z6, Z78, and ORA136 strains (carrying PncA L159P, PncA C138R and PanD E126* mutations, respectively) showed MICs of 800 μg/mL, thereby confirming the discriminatory capability of the method. This finding suggests that PZA DST at neutral pH may offer improved correlation with treatment outcome compared to the current PZA DST at acidic conditions. In this study, the use of fluorescence-based readings in the broth microdilution method for PZA DST in *M. tuberculosis* proved to be both cost-effective and user-friendly.

Although PZase activity is a major determinant of PZA susceptibility, its presence alone does not fully predict MIC levels. The observed MIC variability among PZase-positive strains supports the need for phenotypic testing in addition to genotypic assays. Furthermore, this method may allow detection of subtle resistance phenotypes and support future efforts to define clinically relevant PZA breakpoints. This approach aligns with existing DST frameworks used for other TB drugs and offers the potential for broader implementation in research and clinical settings. Ultimately, standardized MIC testing at neutral pH could enhance the accuracy of PZA resistance detection, reduce false resistance calls, and support more effective treatment decisions. This method could also serve as a platform for defining critical concentrations and interpretive categories [susceptible (S), intermediate (I), and resistant (R)] specific to PZA, advancing global TB resistance surveillance.

In conclusion, this study presents a standardized broth microdilution method at neutral pH (6.8) as a reliable and reproducible approach for determining PZA MICs in *M. tuberculosis*. By addressing key limitations of current acidic pH testing, including false resistance and poor reproducibility, this method enhances the accuracy of PZA susceptibility assessment. The results support its potential use in clinical and surveillance settings. Future large-scale validation using wild-type clinical isolates is essential to define a reliable PZA critical concentration and establish standardized interpretive categories (S/I/R), ultimately improving the clinical utility of PZA in TB treatment.

## Data Availability

The raw data supporting the conclusions of this article will be made available by the authors, without undue reservation.
