# Supplementary material for: Determining the minimum inhibitory concentrations of pyrazinamide against Mycobacterium tuberculosis clinical isolates at a neutral pH of 6.8 using the broth microdilution method
Source: Front Microbiol. 2025 Oct 21;16:1688772. doi: 10.3389/fmicb.2025.1688772 (PMC12584017; doi:10.3389/fmicb.2025.1688772)

**Figure S1**. Concordance between turbidity and fluorescence-based MIC determination for PZA in *M. tuberculosis* H37Ra at neutral pH (6.8) using a standard broth microdilution method. Broth microdilution was performed using a defined medium containing various concentration of PZA (0–800 µg/mL). Each well was inoculated with ~1.0-5.0 × 10⁵ CFU/mL of *M. tuberculosis* H37Ra in a final volume of 200 µL. Plates for turbidity readings were sealed with Parafilm to prevent drying and incubated at 37°C until visible growth was observed in the PZA-free wells. Plates for fluorescence detection included an oxygen-sensitive mycobacterial growth indicator and were sealed with aluminum adhesive film as oxygen barrier and prevent drying. Fluorescence was visualized using a mirrored light box with a 410 nm LED source. (A) Turbidity reading on Day 16: robust growth was observed in the PZA-free wells and partial growth at 12.5 µg/mL; no visible growth was detected at 25 µg/mL, corresponding to the PZA MIC. (B) Negative control for turbidity (no inoculum) confirmed no contamination. (C) Fluorescence reading on Day 10: strong signal was visible in the PZA-free well and diminished signal at 12.5 µg/mL; absence of fluorescence at 25 µg/mL (highlighted in yellow) indicated PZA MIC for *M. tuberculosis* H37Ra. (D) Fluorescence control (no inoculum) showed only background signal, confirming specificity of the assay. These representative images demonstrate full agreement between turbidity- and fluorescence-based PZA MIC determination for *M. tuberculosis* H37Ra at neutral pH (6.8) using dry-format PZA DST plate. Results were reproducible across independent replicates.


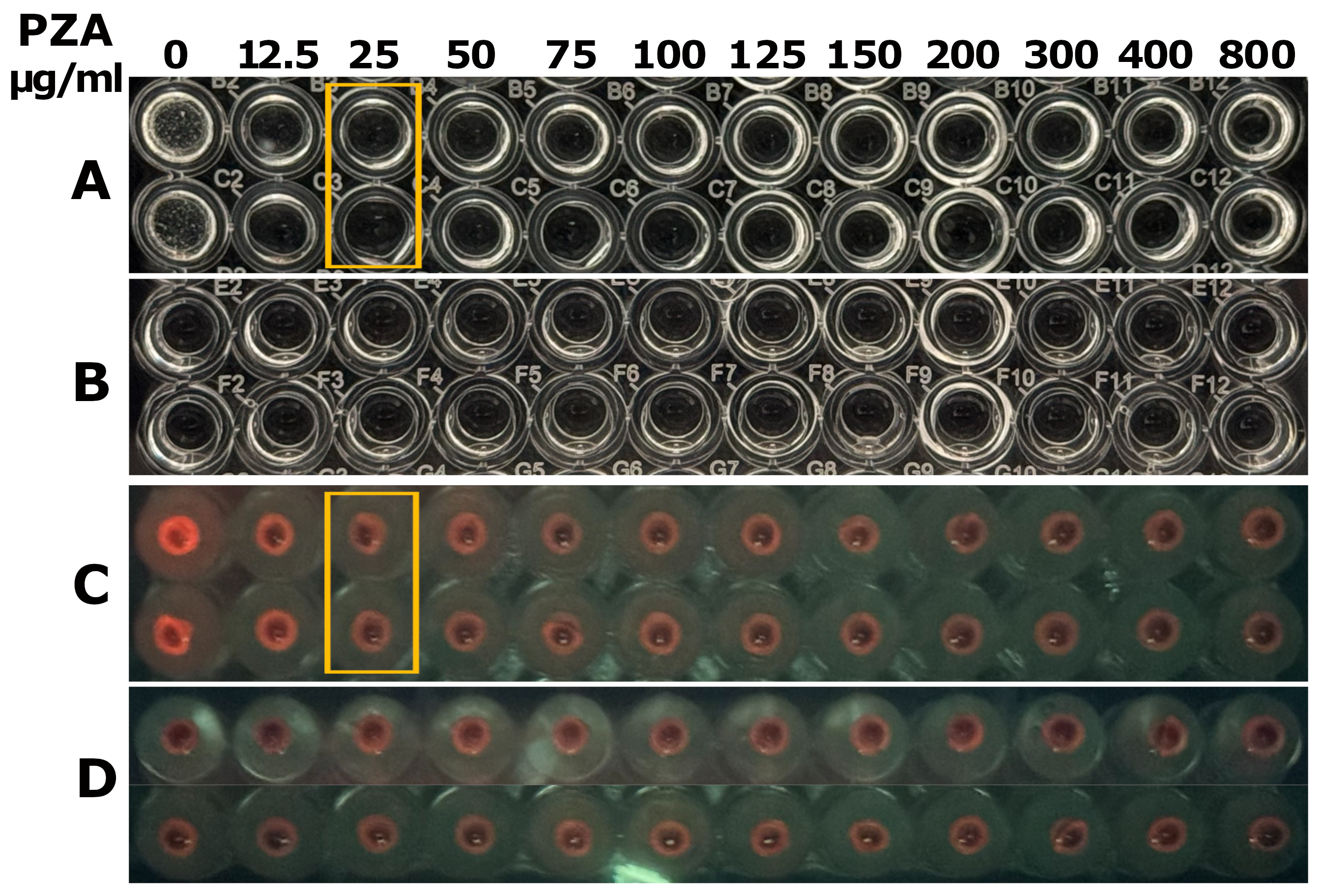


**Figure S2.** Representative examples of Fluorescence-based MIC determination for PZA in *M. tuberculosis* clinical isolates. MIC testing was performed in duplicate using a standard protocol at neutral pH (6.8). Endpoint images were captured when the no-PZA wells exhibited significant fluorescence, using an iPad and a mirrored box with a 410 nm LED source. MIC (indicated with yellow boxes) was defined the lowest concentration of PZA inhibited *M. tuberculosis* visible growth. Strain-specific MIC results are as follows: (A) *M. tuberculosis*, strain HN1135, NR-20786; PZA MIC ≤ 12.5µg/ml. (B) *M. tuberculosis*, strain HN133, NR-20794; PZA MIC = 25µg/ml. (C) *M. tuberculosis*, strain HN1430, NR-18993; PZA MIC = 100µg/ml. (D) *M. tuberculosis*, strain HN2206, NR-20779; PZA MIC ≤ 12.5µg/ml. (E) *M. tuberculosis*, strain HN3171, NR-20776; PZA MIC = 50µg/ml. (F) *M. tuberculosis*, strain HN4687, NR-19033; PZA MIC = 25µg/ml (G) *M. tuberculosis*, strain HN4689, NR-19035; PZA MIC = 25µg/ml, (H) *M. tuberculosis*, strain H37Rv; PZA MIC = 25µg/ml, (I) *M. tuberculosis*, strain Z6; PZA MIC = 800µg/ml, (J) Uninoculated medium control (baseline fluorescence across all wells) showed no visible change in signal by the endpoint (day 20).


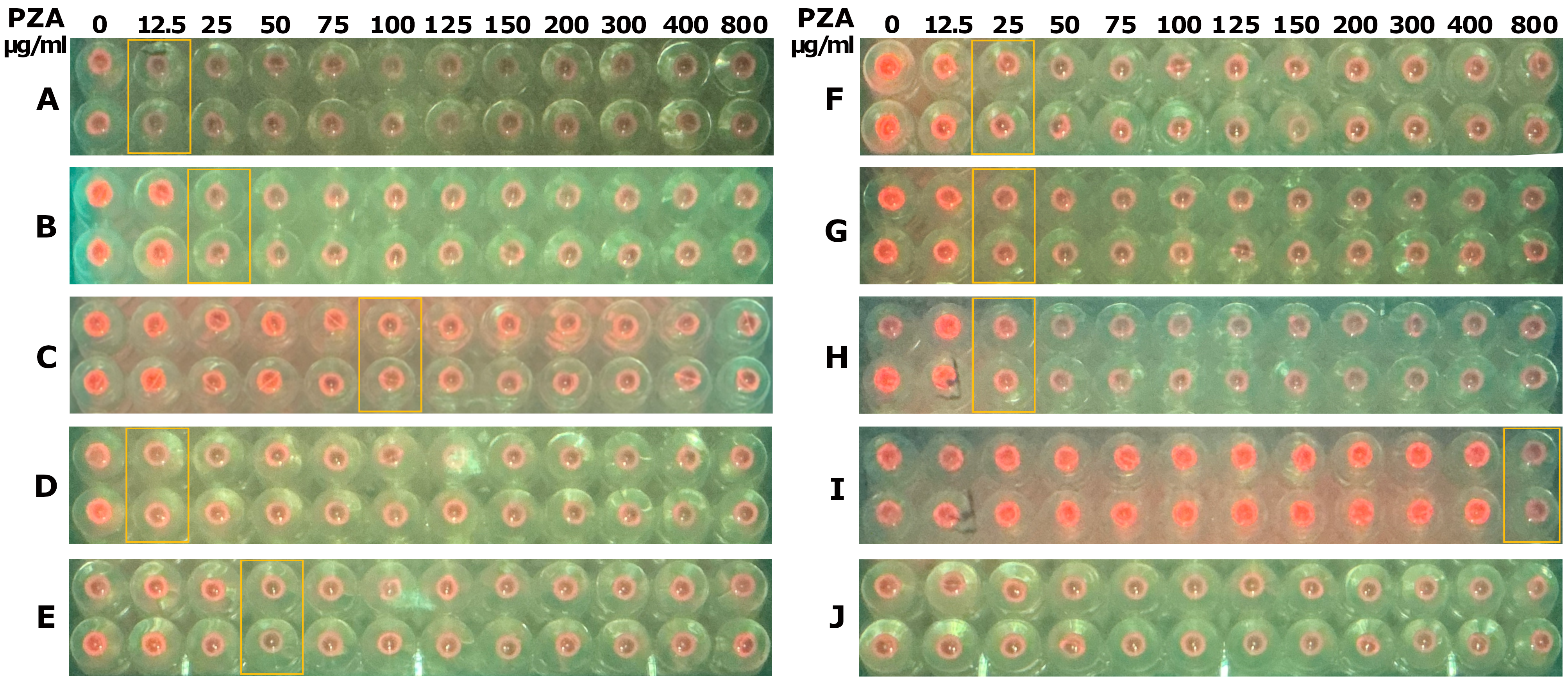

Supplement: Supplementary file 1 [file Data_Sheet_1.docx]
